# *SOD1* and *CAT* gene expressions in stray and owned animals

**DOI:** 10.17221/12/2025-VETMED

**Published:** 2025-08-30

**Authors:** Gonca Sonmez, Mehmet Cevat Temizkan, Muhammed Hudai Culha

**Affiliations:** ^1^Department of Genetics, Faculty of Veterinary Medicine, Selcuk University, Konya, Turkiye; ^2^Department of Laboratory and Veterinary Health, Yozgat Bozok University, Yozgat, Turkiye

**Keywords:** antioxidant, *Canis lupus familiaris*, *Felis catus*, gene expression

## Abstract

Dogs and cats are the most common companion animals in urban environments. Some dogs and cats live in stable home settings, receiving regular care. However, stray dogs and cats are exposed to chronic stress due to life on the streets or in shelters. Stress is correlated with oxidative stress. The dysregulation of redox balance can lead to the accumulation of reactive oxygen species, which promote cellular and molecular damage. In this study, the blood samples were collected from 150 female animals (90 dogs and 60 cats) to isolate peripheral blood mononuclear cells (PBMCs). Total RNA was extracted from PBMCs and subjected to reverse transcription. The expression levels of *SOD1* and *CAT* were quantified using quantitative real-time polymerase chain reaction. Compared with those in stray animals, the *SOD1* expression levels were significantly higher (*P* < 0.01), and the *CAT* expression levels were non-significantly lower in owned animals. These findings suggest that the expression of antioxidant defence genes varied between owned and stray animals. Thus, oxidative stress regulation is dependent on environmental conditions and lifestyle in companion species.

Cats and dogs are the most common domesticated animal species that live with humans in urban environments. Owned animals reside under the care of their owners in stable household settings with access to adequate nutrition and regular veterinary care. In contrast, stray animals spend their lives on the streets or in shelters and are often exposed to unpredictable and adverse conditions. Empirical data are not available to validate the assumption of stray animals being at a high risk of exposure to elevated stress levels due to environmental instability and irregular access to food and shelter (Temizkan and Sonmez 2022). Stress is reported to induce oxidative stress in mammals, especially in metabolically active tissues, such as the skeletal muscle, cardiac muscle, liver, and circulating blood cells (Puppel et al. 2015). The adverse effects of oxidative stress are mediated by reactive oxygen species (ROS), which are generated during aerobic metabolism and can damage lipids, proteins, and DNA. Intricate antioxidant defence systems counteract the effects of ROS. Previous studies have reported that *SOD1* and *CAT* mitigate oxidative damage through their antioxidant activity (Ciani et al. 2021).

SOD, which is universally expressed in aerobic organisms, catalyses the dismutation of superoxide anions (O_2_^–^) into hydrogen peroxide (H_2_O_2_), alleviating oxidative burden. Among the SOD isoforms, SOD1 is primarily localised in the cytosol with a minor fraction localised in the mitochondrial intermembrane space (Sturtz et al. 2001). Thus, SOD1 has a critical role in maintaining redox balance in both cytoplasmic and mitochondrial compartments (Ciani et al. 2021).

The hydrogen peroxide generated by SOD1 activity is further detoxified by peroxidases, such as CAT (Bafana et al. 2011). CAT, a tetrameric haeme-containing enzyme predominantly located in peroxisomes, catalyses the rapid decomposition of hydrogen peroxide into water and molecular oxygen (Srivastava et al. 2012). Thus, CAT has essential functions in metabolically and reproductively active tissues.

The dysregulation of the expression or activity of enzymatic antioxidants can result in oxidative damage. For example, SOD upregulation without concurrent CAT activity upregulation can lead to hydrogen peroxide accumulation and genomic instability, which can be mitigated through CAT-mediated detoxification (Amstad et al. 1991). CAT is involved in hormone signalling and apoptosis regulation (Khan et al. 2020).

Toxicological studies and studies examining chemical exposure have evaluated the expression patterns of oxidative stress-related genes in animal models (Girolami et al. 2021; Zhong et al. 2021). However, studies comparing the expression patterns of these genes between stray and domestic animals have not been performed. This study aimed to evaluate the differential expression of *SOD1* and *CAT* genes in domestic and stray cats and dogs. Animals exposed to harsh environmental conditions, such as stray animals, were hypothesised to exhibit upregulated expression of these antioxidant genes when compared with owned animals.

## MATERIAL AND METHODS

### Animals

This study was performed with 150 female animals [90 dogs (45 owned dogs and 45 stray dogs) and 60 cats (30 owned cats and 30 stray cats)]. The characteristics of the animals were as follows: age: 1–3 years; body weight: 25–45 kg (dogs) or 3–5 kg (cats). The peripheral blood sample (3 ml) was collected from each animal via cephalic venipuncture. To avoid potential physiological alterations, animals were not allowed to engage in physical activity before sampling. The blood samples of owned animals were collected during routine visits to private veterinary clinics after obtaining written informed consent from the owners. Meanwhile, the blood samples of stray animals were obtained during standard veterinary examinations at municipal shelters with institutional permission. According to the shelter records, all stray animals had been born and continuously housed in shelter environments.

### Quantitative real-time polymerase chain reaction (RT-qPCR) analysis

The blood samples were collected into tubes containing the anticoagulant ethylenediaminetetraacetic acid and centrifuged at 2 000 × *g* and 4 °C for 15 minutes. The leukocytes and platelets (in the buffy coat layer) were isolated, resuspended in 10 ml of red blood cell (RBC) lysis buffer [10 mM Tris (hydroxymethyl) aminomethane and 140 mM ammonium chloride (NH_4_Cl)], and incubated on a shaker at room temperature for 15 min to lyse the remaining RBCs. Next, the samples were centrifuged under the same conditions. The pellet containing peripheral blood mononuclear cells (PBMCs) was immediately frozen in liquid nitrogen and stored at −80 °C until RNA extraction. Total RNA was extracted using TRI Reagent (Sigma, Chicago, USA), following the manufacturer’s instructions. Genomic DNA was digested using DNase I (Thermo Fisher Scientific, Waltham, MA, USA). The concentration and purity of RNA were assessed using a NanoDrop ND2000 spectrophotometer. The RNA integrity was verified using 1% agarose gel electrophoresis. Equal concentrations of RNA (400 ng/μl) were standardised using diethyl pyrocarbonate-treated water. Complementary DNA (cDNA) was synthesised using the iScript^TM^ cDNA synthesis kit (Bio-Rad Laboratories, Hercules, CA, USA), following the manufacturer’s instructions.

The mRNA sequences of the target genes were retrieved from the GenBank database of the National Center for Biotechnology Information (NCBI). The primer pairs were designed using Oligo v7.60 (Molecular Biology Insights, USA) and NCBI Primer-BLAST tools. Care was taken to avoid the formation of heterodimers and self-dimers during primer selection, following the methods of Wang and Seed (2003). RT-qPCR analysis was performed using the Roche LightCycler Nano System with the iTaq^TM^ Universal SYBR^®^ Green qPCR kit (Bio-Rad Laboratories, Hercules, CA, USA). A 20-μl reaction mixture containing 4 μl of cDNA, 100 pM of forward primer, 100 pM of reverse primer, 10 μl of SYBR Green master mix, and nuclease-free water (to make up the final volume) was prepared. The PCR conditions were as follows: 98 °C for 180 s (initial denaturation), followed by 35 cycles of denaturation at 95 °C for 15 s, annealing at the gene-specific temperature shown in Table 1 for 30 s, and extension at 72 °C for 30 seconds. Melting curve analysis was performed at 60–95 °C at an incremental rate of 0.1 °C/10 s to verify the specificity of amplification. All reactions were performed in triplicate for each sample. Visualising band sizes further confirmed amplicon specificity on a 1% agarose gel. *YWHAZ* (tryptophan 5-monooxygenase activation protein zeta polypeptide) and *TBP* (TATA-box binding protein) served as the reference genes for cats (Peters et al. 2007) and dogs (Kessler et al. 2009), respectively. The primer sequences for the housekeeping genes were adopted from previous studies (Temizkan and Sonmez 2022; Temizkan et al. 2023).

**Table 1:** Primer sequences, amplicon lengths, and annealing temperatures

Animal group	Gene	Primer sequence (5'–3')	Amplicon length	Annealing temperature	NCBI Accession No.
Dog/cat	*SOD1* – F	GGTATCAGGAACCATTACAGG	dog: 289 cat: 289	dog: 55 °C cat: 60 °C	dog: NM_001003035.1 cat: XM_006935922.4
	*SOD1* – R	CAAGTCATCTCGTTTCTCGT			
Dog/cat	*CAT* – F	TTCTTGTTCAGTGATCGAGGG	dog: 160 cat: 160	dog: 56.2 °C cat: 55.8 °C	dog: NM_001002984.1 cat: XM_003993157.5
	*CAT* – R	CTGCGTCTTCAACAGAAAGG			
Dog	*TBP* – F	AGGAAGACAACGTAATGGCTGT	212 bp	59 °C	XM_038684469
	*TBP* – R	TTCTCTACGCAGAAGGAAGACC			
Cat	*YWHAZ* – F	GATGGCTCGAGAATACAGAGAGA	167 bp	56 °C	XM_006943327
	*YWHAZ* – R	CAACCTCAGCCAAGTAACGATAG			

### Statistical analysis

The relative gene expression levels were calculated using the 2^−ΔΔCt^ method (Livak and Schmittgen 2001) to determine the fold change in gene expression between owned and stray animal groups. *TBP* and *YWHAZ* were used as reference genes for dogs and cats, respectively. The normal distribution of the data was assessed using the Anderson–Darling test, which is suitable for small sample sizes and sensitive to deviations from a normal distribution. Means between the groups were compared using an independent samples *t*-test in SPSS v22 (IBM, USA). Differences were considered significant at *P* < 0.01.

### Ethics statement

The Local Ethics Committee approved the experimental protocol of this study for Animal Experiments of Erciyes University (Approval No.: 22/210).

## RESULTS AND DISCUSSION

The primer efficiencies were determined using a standard curve generated from serially diluted pooled cDNA samples. The efficiency values, linear equations, and coefficient of determination (*R*^2^) for each primer pair are shown in Table 2. Only primers with efficiency values in the range of 1.9–2.2 were used for RT-qPCR analyses to ensure amplification reliability. The expression of *SOD1* in owned dogs was significantly (1.27-fold) higher than that in stray dogs (*P < *0.01).

**Table 2:** Primer efficiency

Animal group	Gene	Formula	*R* ^2^	Efficiency
Dog	*SOD1*	*y* = –3.36*x* + 21.38	0.99	1.99
Cat	*SOD1*	*y* = –3.38*x* + 25.24	0.99	1.98
Dog	*CAT*	*y* = –3.30*x* + 20.84	0.99	2.01
Cat	*CAT*	*y* = –3.05*x* + 27,02	0.94	2.13
Dog	*TBP*	*y* = –3.42*x* + 23.29	0.99	1.96
Cat	*YWHAZ*	*y* = –3.48*x* + 23.96	0.99	1.94

**Figure 1:**
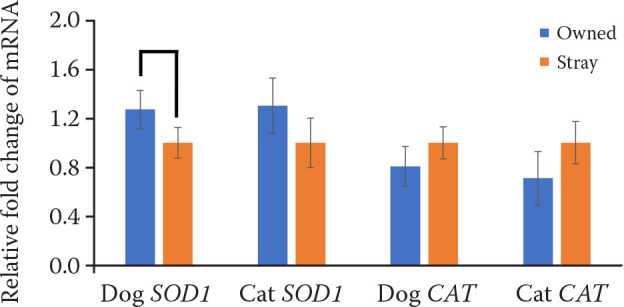
Gene expression profiles of antioxidant genes

Similarly, the expression of *SOD1* in owned cats was non-significantly (1.30-fold) higher than that in stray cats. The expression of *CAT* in owned cats and dogs was non-significantly lower than that in stray cats and dogs, respectively. These findings indicate that the *SOD1* and *CAT* expression patterns were similar between owned and stray animals (Table 3; Figure 1).

**Table 3:** Gene expression profiles of antioxidant genes

Animal group	Group	*N*	2^−ΔΔCt^	Standard error	*P-*value
Dog	owned *SOD1*	45	1.27	0.16	0.006*
	stray *SOD1*	45	1.00	0.13	
	owned *CAT*	45	0.80	0.16	0.074
	stray *CAT*	45	1.00	0.13	
Cat	owned *SOD1*	30	1.30	0.22	0.306
	stray *SOD1*	30	1.00	0.20	
	owned *CAT*	30	0.71	0.22	0.085
	stray *CAT*	30	1.00	0.17	

*P < 0.01

The antioxidant enzymes SOD1 and CAT play crucial roles in cellular defence against ROS, protecting biomolecules against oxidative damage and maintaining redox homeostasis. Various physiological and environmental factors, including stress, infection, nutritional status, and exposure to external stressors, influence the expression of these genes. Based on the distinct living conditions of owned and stray animals, the *SOD1* and *CAT* expression levels serve as useful molecular markers for evaluating oxidative stress responses to differential environmental and lifestyle conditions.

Previous studies have reported that *SOD1* mutations lead to neurodegenerative diseases, especially canine degenerative myelopathy, in dogs (Awano et al. 2009; Wininger et al. 2011). Additionally, the expression of *SOD1* and *CAT* varies with nutritional interventions, stress exposure, and metabolic changes. For example, antioxidant supplementation, melatonin treatment, and dietary modifications upregulate the expression of *SOD1* and *CAT* (Qi et al. 2021; Salavati et al. 2021). Dogs exposed to acute inflammatory stimuli, such as lipopolysaccharide, exhibited *CAT* downregulation in hepatic tissues (Higgins et al. 2003). These findings highlight the dynamic regulation of antioxidant genes in response to both intrinsic and extrinsic factors.

Limited studies have examined the expression of oxidative stress-related genes in cats. Current cat studies focus on reproductive physiology, renal pathology, or *in vitro* toxicological responses. For example, Braun et al. (2020) reported phase-specific differences in *SOD1* and *CAT* expression during feline reproductive cycles. Alborough et al. (2020) reported that trace element deficiencies, especially copper and zinc deficiencies, are associated with *SOD1* downregulation in the kidneys of cats with chronic interstitial nephritis. Additionally, methimazole-induced oxidative stress in feline kidney epithelial cells upregulated *SOD1* and *CAT*, highlighting their responsiveness to increased ROS production (Girolami et al. 2021). These findings underscore the importance of considering physiological context when interpreting gene expression data in felines.

In this study, *SOD1* expression in owned dogs was significantly higher than that in stray dogs (*P* < 0.01). Although a similar trend was observed in cats, the difference was not significant. In contrast, *CAT* expression was non-significantly downregulated in owned dogs and cats. These findings suggest that *SOD1* is more sensitive to environmental modulation than *CAT* and that the gene expression responses are subtle and potentially influenced by underlying biological complexity.

One plausible explanation for the limited differences observed in this study is the life history of the stray animals. Animals continuously exposed to outdoor conditions since birth may have developed physiological adaptations to persistent environmental stressors, leading to stable antioxidant responses and baseline gene expression. Early-life environmental exposures can program long-term stress responses by regulating the hypothalamic-pituitary-adrenal axis, resulting in reduced physiological sensitivity to stress in adulthood (Liu et al. 1997).

The adaptation hypothesis is further substantiated by the concept of stress tolerance acquired through long-term environmental habituation. Prolonged exposure to challenges, such as inconsistent feeding, temperature fluctuations, and unstable social dynamics, may alter homeostatic regulation. These alterations may be mediated through transcriptional regulation and epigenetic mechanisms, including DNA methylation and histone modification. Epigenetic modifications modulate stress responses across the lifespan (Feil and Fraga 2012). Thus, the gene expression profiles of animals that have lived as strays since birth may reflect a physiologically adapted and compensated state rather than an acute response to ongoing environmental stressors.

Although owned animals are assumed to experience decreased stress, they may encounter episodic stressors, such as confinement, reduced physical activity, or medical interventions, which are reported to increase physiological stress markers in dogs (Beerda et al. 1997). This can explain the upregulated expression of *SOD1* in owned dogs in this study. However, the upregulation of *SOD1* was not observed in cats, which can be attributed to species-specific differences or sample size limitations. Additionally, the mRNA levels may not accurately reflect functional antioxidant capacity, as post-transcriptional and post-translational mechanisms can significantly influence protein activity.

*SOD1* expression was upregulated in owned dogs. However, the overall expression profiles across groups suggest a complex interplay of environmental exposure, physiological adaptation, and possibly epigenetic regulation.

This study improved our understanding of oxidative stress responses in companion animals. The findings of this study suggest the need to consider early-life environment and long-term adaptation when interpreting gene expression data. Future studies should include broad biomarker panels, enzyme activity assays, and controlled experimental designs that distinguish between animals born and raised in different environments. Additionally, longitudinal studies may provide valuable insights into the cumulative impact of environmental conditions on the antioxidant defence system.

In this study, the expression levels of *CAT* and *SOD1* were evaluated in female-owned and stray animals (60 cats and 90 dogs). *SOD1* expression in owned dogs was significantly higher than that in stray dogs (*P* < 0.01). Meanwhile, *SOD1* expression was non-significantly upregulated in owned cats. In contrast, *CAT* gene expression was non-significantly downregulated in both owned dogs and cats. Thus, the expression patterns of these antioxidant genes were broadly similar between owned and stray animals. These results suggest that ownership status alone is not a strong determinant of oxidative stress-related gene expression alterations and highlight the need to consider additional factors, such as environmental adaptation, early-life conditions, and physiological resilience, when interpreting antioxidant responses in companion animals.
